# Polyunsaturated Fatty Acid Composition of Cerebrospinal Fluid Fractions Shows Their Contribution to Cognitive Resilience of a Pre-symptomatic Alzheimer’s Disease Cohort

**DOI:** 10.3389/fphys.2020.00083

**Published:** 2020-02-14

**Authors:** Alfred N. Fonteh, Matthew Cipolla, Abby J. Chiang, Sarah P. Edminster, Xianghong Arakaki, Michael G. Harrington

**Affiliations:** Neurosciences, Huntington Medical Research Institutes, Pasadena, CA, United States

**Keywords:** Alzheimer’s disease, cerebrospinal fluid, polyunsaturated fatty acids, mass spectrometry, cognition, resilience, brain-derived nanoparticles

## Abstract

Alzheimer’s disease (AD) pathology is characterized by an early and prolonged decrease in the amyloid peptide (Aβ) levels concomitant with a later increase in phospho-tau concentrations in cerebrospinal fluid (CSF). We propose that changes in lipid metabolism can contribute to the abnormal processing of Aβ_42_ in AD. Our aim was to determine if polyunsaturated fatty acid (PUFA) metabolism can differentiate pre-symptomatic AD from normal aging and symptomatic AD. Using neuropsychology measures and Aβ_42_/T-tau in cerebrospinal fluid (CSF), we classify three groups of elderly study participants: cognitively healthy with normal Aβ_42_/T-tau (CH-NAT), cognitively healthy with pathological Aβ_42_/T-tau (CH-PAT), and AD individuals. We determined the size distribution and the concentration of CSF particles using light scattering and quantified PUFA composition in the nanoparticulate (NP) fraction, supernatant fluid (SF), and unesterified PUFA levels using gas chromatography combined with mass spectrometry. Four PUFAs (C20:2n-6, C20:3n-3, C22:4n-6, C22:5n-3) were enriched in NP of AD compared with CH-NAT. C20:3n-3 levels were higher in the NP fraction from AD compared with CH-PAT. When normalized to the number of NPs in CSF, PUFA levels were significantly higher in CH-NAT and CH-PAT compared with AD. In the SF fractions, only the levels of docosahexaenoic acid (DHA, C22:6n-3) differentiated all three clinical groups. Unesterified DHA was also higher in CH-NAT compared with the other clinical groups. Our studies also show that NP PUFAs in CH participants negatively correlate with CSF Aβ_42_ while C20:4n-6, DHA, and n-3 PUFAs in the SF fraction positively correlate with T-tau. The profile of PUFAs in different CSF fractions that correlate with Aβ_42_ or with T-tau are different for CH-NAT compared with CH-PAT. These studies show that PUFA metabolism is associated with amyloid and tau processing. Importantly, higher PUFA levels in the cognitively healthy study participants with abnormal Aβ_42_/T-tau suggest that PUFA enhances the cognitive resilience of the pre-symptomatic AD population. We propose that interventions that prevent PUFA depletion in the brain may prevent AD pathology by stabilizing Aβ_42_ and tau metabolism. Further studies to determine changes in PUFA composition during the progression from pre-symptomatic to AD should reveal novel biomarkers and potential preventive approaches.

## Introduction

A hallmark of AD pathology is the formation of neurotoxic amyloid plaques and increased phosphorylation of total tau (T-tau) (Skoog et al., 2015). Lower levels of Aβ_42_ and higher levels of T-tau are found in cerebrospinal fluid of AD subjects leading to a lower Aβ_42_/T-tau ratio in AD compared with cognitively healthy (CH) study participants (Blennow et al., 2001; Harrington et al., 2013). These changes in Aβ_42_/T-tau have been recognized as a sensitive biomarker of AD (Skoog et al., 2015). However, it is now clear that some CH individuals have Aβ_42_/T-tau ratios similar to AD, suggesting that Aβ_42_/T-tau ratio may not distinguish some asymptomatic CH subjects from AD. With a similar Aβ_42_/T-tau for some CH and AD subjects, a major question that arises is whether there are other defining biochemical differences between these clinical populations. Secondly, what accounts for the cognitive resilience (Negash et al., 2013; Boros et al., 2017; Aiello Bowles et al., 2019) or reserve (Cummings et al., 1998; Persson et al., 2017; Menardi et al., 2018) in some elderly persons who have abnormal Aβ_42_? Since amyloid precursor protein (APP) is a membrane-bound protein influenced by membrane biophysics and trafficking (Askarova et al., 2011; Tan and Gleeson, 2019), we propose that changes in the lipid environment in post-mitotic neurons may influence the processing and formation of amyloidogenic or non-amyloidogenic peptides.

Fatty acyls are a major component of membrane lipids and can influence AD pathology in several ways. Palmitoylation of APP influences Aβ_42_ formation (Bhattacharyya et al., 2013; Andrew et al., 2017), and the ratios of different fatty acids in gangliosides have been shown to influence aggregation of Aβ_42_ (Oikawa et al., 2009; Oikawa et al., 2015). The fatty acyl composition of cellular membranes contributes to their physical properties and the activities of transmembrane proteins. For example, the amount of cholesterol and the ratios of saturated to unsaturated fatty acids known to influence the biophysical properties of membranes have been shown to increase the interaction of APP with beta-secretase in early AD (Kametaka et al., 2003; Marlow et al., 2003; Avila-Munoz and Arias, 2015). In rodent studies, high saturated fat diets favor amyloid deposition, while PUFA (DHA) supplemented diets decrease amyloid accumulation and attenuate glial cell activation (Oksman et al., 2006). In addition to membrane fluidity, PUFAs are associated with cognitive function and memory (Cardoso et al., 2016). Supplementation with DHA/EPA maintains levels of pro-resolving or neuro-protecting mediators (Serhan et al., 2015; Thau-Zuchman et al., 2019), suggesting a protective role of PUFAs in post-mitotic neurons. Oxidation of PUFA by an enzyme or non-enzymatic pathways generates inflammatory and toxic products, and these are increased in AD (Montine and Morrow, 2005; Lukiw and Bazan, 2008; Grimm et al., 2016). Neuroinflammatory pathways are associated with cognitive decline, and PUFAs are the source of several lipid mediators of inflammation that are altered in AD (Pomponi et al., 2008). Moreover, several enzymes that hydrolyze PUFAs, such as phospholipases A_2_, are altered in AD (Stephenson et al., 1996; Farooqui and Horrocks, 1998; Sanchez-Mejia and Mucke, 2010; Fonteh et al., 2013).

With these important associations with AD pathology, we examined PUFA composition in CH individuals with normal Aβ_42_/T-tau (CH-NAT) compared with elderly CH study participants with pathological Aβ_42_/T-tau (CH-PAT) and with AD (Harrington et al., 2013). We show that PUFA metabolism distinguishes pre-symptomatic AD from symptomatic AD, suggesting that PUFAs may contribute to the cognitive resilience of the pre-symptomatic population. PUFA levels in CH study participants negatively correlated with Aβ_42_ and positively correlated with T-tau, suggesting that PUFAs contribute to the metabolism of these peptides. Our studies suggest that early changes in PUFA metabolism may contribute to AD pathology by disrupting brain membrane structures and initiating the abnormal processing and transport of denatured proteins. Therefore, detection of early PUFA changes in the brain may reveal mechanisms that account for pre-symptomatic AD progression and can be explored to prevent AD pathology.

## Materials and Methods

### Recruitment and Classification of Study Participants

All study protocols and consent forms were approved by the Institutional Review Board of the Huntington Memorial Hospital, Pasadena, CA, United States (HMH-99-09). Written informed consent was obtained from all study participants. Demographic data, medical, and diagnostic procedures have been described (Harrington et al., 2013). Participants were included (Table 1) if they were classified as CH-NAT, CH-PAT, or with clinically probable AD (McKhann et al., 2011).

**TABLE 1:** Demographic data, CSF chemistry, APOE genotype, and plasma lipid levels in the clinical subgroups.

Parameters	CH-NAT (*n* = 36)	CH-PAT (*n* = 34)	AD (*n* = 25)

Female (%)	61	62	52

	mean ± SD		
Age (years)	76.4 ± 7.1	78.0 ± 6.5	76.0 ± 9.1
**CDR (mean)**			
BMI^#1^	1.5 ± 0.8	1.8 ± 0.7	1.6 ± 0.7
Education^#2^	6.3 ± 1.8	6.0 ± 2.3	3.8 ± 2.5
Aβ_42_ (pg/ml)	916 ± 211	518 ± 237	460 ± 196
T-tau (pg/ml)	197 ± 67	353 170	517 ± 205
Aβ_42_/T-tau	5.1 ± 1.8	1.6 ± 0.6	1.0 ± 0.5
*APOE risk*^#3^	3.17 ± 1.1	2.82 ± 1.3	3.52 ± 1.1
CSF protein (μg/ml)	402 ± 16	396 ± 15	358 ± 12
Triglyceride	106 ± 52	106 ± 46	102 ± 39
Total cholesterol	187 ± 29	179 ± 33	183 ± 31
HDL	64 ± 15	61 ± 18	62 ± 15
LDL	111 ± 28	104 ± 25	110 ± 27

*^#1^BMI – Underweight = 0, Normal weight = 1, Overweight = 2, Obese = 3. ^#2^Education – Less than HS = 0; HS Diploma = 1; Technical or trade school = 2; Some college = 3; 2-year college = 4; College, more than 2-year degree but not a 4-year degree = 5, 4-year college degree = 6; Some post-graduate = 7; ^#3^*APOE* Genotype – increasing risk from E4 (2/2 = 0; 2/3 = 1; 3/3 = 2; 2/4 = 3; 3/4 = 4; 4/4 = 5).*

### APOE Genotype

mRNA from peripheral blood lymphocytes was used for APOE genotyping and was performed using a polymerase chain reaction mixture of specific primers for E2, E3, and E4 (Calero et al., 2009). To determine the effects of ApoE on PUFA metabolism, we pegged each participant based on their risk from ApoE4: non-carriers of ApoE4 allele (E2/3 = 1, E3/E3 = 2) were grouped together while ApoE4 carriers (E2/E4 = 3, E3/E4 = 4) and homozygous ApoE4 (E4/E4 = 5) formed different groups for high and highest risks, respectively.

### CSF Collection, Total Protein, Aβ_42_, and T-tau Measures

Cerebrospinal fluid was collected between 8:00 a.m., and 10:00 am after an overnight fast. Total protein concentrations, Aβ_42_ and T-tau assays, were performed using CSF aliquots after a single thaw as previously described (Harrington et al., 2013).

### Materials

HPLC grade water, chloroform, methanol, formic acid, and anhydrous acetonitrile required for lipid extraction were purchased from VWR (West Chester, PA, United States). Hydrochloric acid and butylated hydroxytoluene (BHT) were purchased from Sigma (St. Louis, MO, United States). Linoleic Acid-d_4_, α-Linolenic Acid-d_14_, Arachidonic Acid-d_8_, Eicosanoic Acid-d_3_, Eicosapentaenoic Acid-d_5_, Docosanoic Acid-d_43_, and Docosahexaenoic Acid-d_5_ (Avanti Polar Lipids, Alabaster, AL, United States) were used as internal standards.to monitor PUFA extraction recovery and for quantification. Non-deuterated FA standards containing a mixture of 50 free fatty acids were purchased from NuChek Prep (Elysian, MN, United States). Pentafluorobenzyl bromide (PFBBr) from Thermo Fisher Scientific (Bellafonte, PA, United States) and NN-diisopropylethanolamine (DIPE) from Sigma-Aldrich were used for synthesizing PFBBr-derivatives of PUFAs.

### CSF Fractionation and Fatty Acid Extraction

CSF supernatant fluid (SF) or nanoparticle (NP) fractions were obtained by centrifugations as previously described (Harrington et al., 2009). After the addition of a deuterated fatty acid standard cocktail (100 ng each), fatty acids were extracted from each fraction using a modified Bligh and Dyer procedure (Bligh and Dyer, 1959).

### Acid Hydrolysis of Extracted Lipids

We obtained the PUFA composition of each CSF fraction by first hydrolyzing aliquots (250 ul) of the lipid extracts, as previously described (Aveldano and Horrocks, 1983). The fatty acid-enriched CHCl_3_ extract was washed using 2 mL NaCl (1 M) before the addition of 1 mL CH_3_OH containing 0.1 mg/mL BHT.

### Derivatization of Lipid Extracts

Hydrolyzed fatty acids from the NP or SF fractions were dried under a stream of N_2_ and then converted to pentafluorobenzyl esters using a mixture of PFBBr in acetonitrile solution (1:19 v/v, 50 μL) and DIPE in acetonitrile solution (1:9 v/v, 50 μL) for 20 min at 45°C with vortexing every 10 min (Quehenberger et al., 2008). After the removal of reagents using N_2_ drying, derivatized FAs were extracted with 1 mL hexane (x2) (Chilton et al., 1993). The combined hexane extract was dried under N_2_, and the derivatized fatty acids were dissolved in 50 μL dodecane before transfer into GC-MS vials.

### GC-MS Analyses of Derivatized Fatty Acids

We obtained fatty acid levels in CSF fractions using GC negative ion chemical ionization MS (Chilton et al., 1993). Single ion monitoring was used to measure carboxylate ions for deuterated standards and samples. We used the same list for carboxylate ions (m/z) of non-deuterated and deuterated fatty acid standards (Fonteh et al., 2014).

### CSF Nanoparticle (NP) Sizing

Particle size number and distribution in CSF was determined using a NanoSight NS300 instrument (Malvern Panalytical Inc., Westborough, MA, United States). Briefly, freshly collected CSF was centrifuged (3000 RCF, x 3 min), and 1 ml aliquots were frozen. CSF was diluted 10-fold (dd-H_2_O) and continuously infused into the NS300 that had been calibrated with polystyrene beads (30 nm, 100 nm, and 400 nm). The sample temperature was maintained at 25°C, and recordings were obtained at 432 nm for 60 s (x5) with 10 s delay between recordings. The data were acquired and processed using the Nanoparticle Tracking Analysis software (Malvern Panalytical Inc.).

### Data Analyses

MassHunter Workstation Software (Agilent) was used to analyze GC-MS fatty acid data. Calibration and standard curves were obtained using deuterated fatty acid standards All CSF samples and standards were analyzed in replicates, followed by automatic peak integration for most fatty acids.

### Statistical Analyses

ANOVA with Tukey’s Multiple Comparison tests or the Mann–Whitney *U* tests were performed to determine significant differences in fatty acid levels between CH-NAT, CH-PAT, and AD study participants. Spearman’s rank analyses were used to determine the correlation of PUFAs with Aβ_42_ or T-tau. Receiver operating characteristic (ROC curve) was performed to determine if larger or smaller values of fatty acids can classify AD or CH-NAT over the CH-PAT subjects. All statistical analyses were performed using GraphPad Prism software (La Jolla, CA, United States) or MetaboAnalyst software and data were considered significant if adjusted P for false discovery rate was <0.05 (Chong et al., 2018). Briefly, data in an Excel sheet was coded for CH-NAT (=1), CH-PAT (=2), and AD (=3), and the file was converted to tab-delimited text (.txt) prior to import into the MetaboAnalyst Statistical Analysis platform. The data were normalized using globalized logarithm transformation (glog) and then scaled by mean-centering before ANOVA with Tukey *post hoc* analyses (Chong et al., 2019). This data processing resulted in a Gaussian distribution and scaling enabled us to compare PUFA levels that are several orders of magnitude in CSF fractions.

## Results

### Clinical Demographics and AD Risk Factors

The demographic data, AD risk factors, and CSF Aβ_42_, and T-tau levels are shown in Table 1. The AD group was less educated than the other groups, as we and others previously reported (Roe et al., 2008; Harrington et al., 2013). The rank order of CSF Aβ_42_ was CH-NAT>CH-PAT>AD while this was reversed for T-tau. This resulted in a significantly lower CSF Aβ_42_/T-tau in CH-PAT and AD compared with CH-NAT (Table 1). Thus, the Aβ_42_/T-tau pathology distinguished two CH groups, one set with higher Aβ_42_/T-tau (CH-NAT) and another group (CH-PAT) with Aβ_42_/T-tau values similar to AD. We describe this group as a pre-symptomatic AD cohort because 4 years longitudinal follow-up study shows that 25% of these participants progress to MCI or AD while no CH-NAT participants deteriorate in the same period (Harrington et al., 2019).

### Dietary Fatty Acids and AD

We obtained fasting levels of triglycerides, cholesterol, HDL, and LDL to isolate dietary influence on fatty acid metabolism. No differences were detected in each lipid class in our clinical groups (Table 1). Using a validated self-reported Dietary Health Questionnaire (DHQ), energy and consumption, and dietary fatty acid compositions were accessed. No significant differences were measured in the dietary levels of fatty acids by CH-NAT and CH-PAT (Table 2).

**TABLE 2:** Energy and dietary lipid consumption – Cognitively healthy study participants completed an online DHQ, and their lipid consumption was estimated using DietCal (NCI-NIH).

DHQ item	CH_NAT Mean (SEM)	CH_PAT Mean (SEM)
Food energy (kcal)	1824 ± 124	1656 ± 110
Total fat (g)	63 ± 24	55 ± 21
Carbohydrate (g)	244 ± 18	234 ± 16
Protein (g)	65 ± 4	57 ± 4
Cholesterol (mg)	190 ± 14	161 ± 14
Saturated fat (g)	21 ± 2	18 ± 1
Monounsaturated fat – g	23 ± 2	20 ± 2
Polyunsaturated fat – g	14 ± 1	13 ± 1
Fatty acid 20:4 – g	0.09 ± 0.007	0.07 ± 0.008
Fatty acid 20:5 – g	0.03 ± 0.006	0.04 ± 0.008
Fatty acid 22:6 – g	0.06 ± 0.008	0.06 ± 0.01
Fatty acid 10:0 – g	0.4 ± 0.05	0.3 ± 0.04
Fatty acid 14:0 – g	1.9 ± 0.2	1.6 ± 0.2
Fatty acid 16:1 – g (CSFII)	1 ± 0.1	0.9 ± 0.07
Fatty acid 18:2 – g (CSFII)	12.7 ± 0.9	11.3 ± 0.9
Fatty acid 18:3 – g	1.4 ± 0.1	1.2 ± 0.1
Fatty acid 22:5 – g	0.01 ± 0.002	0.01 ± 0.002
18:2 *TRANS* (*trans-*octadecadienoic acid [linolelaidic acid]) – g (NDS-R)	0.5 ± 0.04	0.4 ± 0.04
Dietary fiber – g (CSFII)	21 ± 2	21 ± 2
Total dietary fiber – g (NDS-R)	20 ± 2	20 ± 2
Insoluble dietary fiber – g (NDS-R)	13 ± 1	13 ± 1
Soluble dietary fiber – g (NDS-R)	7 ± 1	7 ± 1

### Fatty Acid Composition of CSF Fractions

**FIGURE 1:**
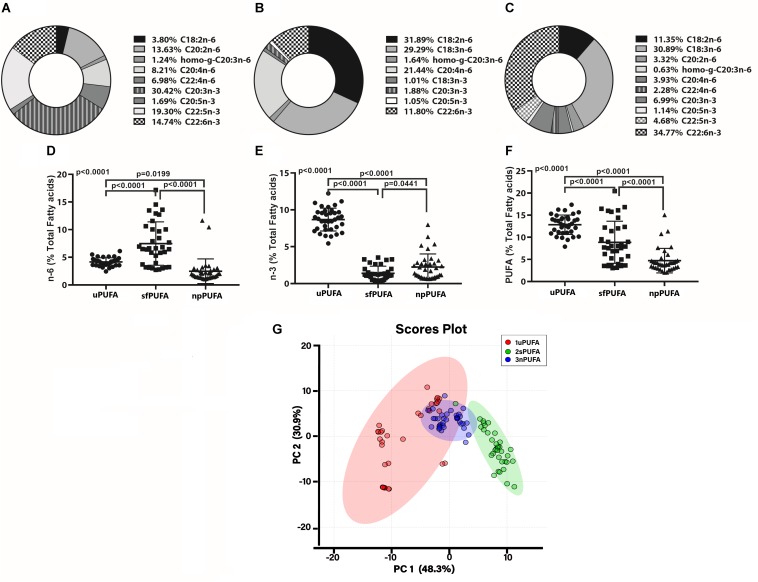
PUFA distribution in CSF fraction – The doughnut graphs show the proportion of n-3 and n-6 PUFAs quantified in the NP fraction of CSF **(A)**, the supernatant fluid fraction **(B)**, or as unesterified fatty acids **(C)**. Comparison of the n-6 PUFA amounts in the three CSF fractions **(D)**, n-3 PUFA in the fractions **(E)**, and total PUFAs **(F)** using 1-way ANOVA with Dunn’s multiple tests showing adjusted *p*-values. PCA of the distribution of PUFA species in the CSF fractions **(G)**.

We next measured PUFA levels in CSF fractions: nanoparticles derived from brain membranes (npPUFA), supernatant fluid representative of interstitial metabolism (sfPUFA), and unesterified PUFA levels (uPUFA) resulting from lipolytic enzyme activities that increase in AD. The proportion of several n-6 and n-3 species varied between the various fractions (Figures 1A–C). Our data show the differential distribution of PUFAs in CSF fractions. The proportion of n-6 PUFAs was highest in the SF fraction (Figure 1D), while n-3 was highest in the unesterified fractions (Figure 1E). Total PUFA was lowest in the NP fraction (Figure 1F). Differences in the distribution of PUFAs in CSF fractions are supported by principal component analyses, showing the interaction of npPUFA with uPUFA but not with sfPUFA (Figure 1G).

### Changes in Fatty Acid Composition in the CSF Fractions

#### PUFA Concentration and Composition in NP Fractions (Figure 2 and Supplementary Table 1)

**FIGURE 2:**
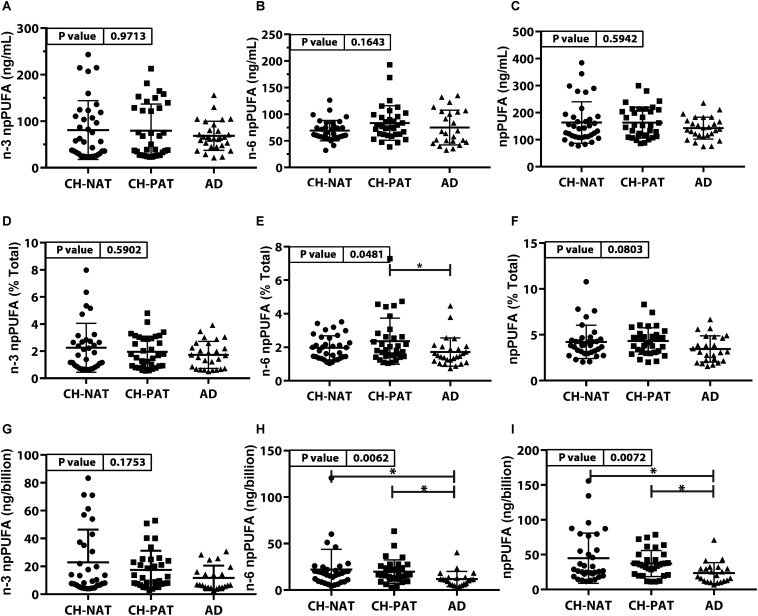
PUFAs in nanoparticles from CH-NAT, CH-PAT, and AD – Scatter plots (mean ± SD) of the concentrations of n-3 npPUFAs **(A)**, n-6 npPUFA **(B)**, and npPUFA **(C)** showing *p*-values determined using 1-way ANOVA and adjusted *p*-values < 0.05 indicated by an asterisk (^∗^). Scatter plots of the proportions of n-3 PUFAs **(D)**, n-6 npPUFA **(E)**, and npPUFA **(F)** showing *p*-values obtained using 1-way ANOVA and adjusted *p*-values < 0.05 indicated by an asterisk (^∗^). Concentrations of npPUFAs per billion nanoparticles for n-3 npPUFAs **(G)**, n-6 npPUFA **(H)**, and npPUFA **(I)** showing *p*-values obtained using 1-way ANOVA and adjusted *p*-values < 0.05 indicated by an asterisk (*).

**FIGURE 3:**
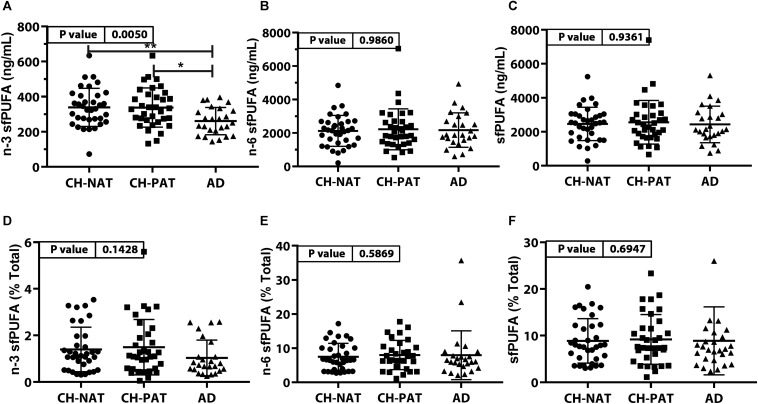
PUFAs in supernatant fluid (sf) – Scatter plots (mean ± SD) of the concentrations of n-3 sfPUFA **(A)**, n-6 sfPUFA **(B)**, and sfPUFA **(C)** showing *p*-values determined using 1-way ANOVA and adjusted *p*-values < 0.05 indicated by an asterisk (*). Plots of the proportions of n-3 sfPUFA **(D)**, n-6 sfPUFA **(E)**, and sfPUFA **(F)** showing *p*-values obtained using 1-way ANOVA and adjusted *p*-values < 0.05 indicated by an asterisk (*).

**FIGURE 4:**
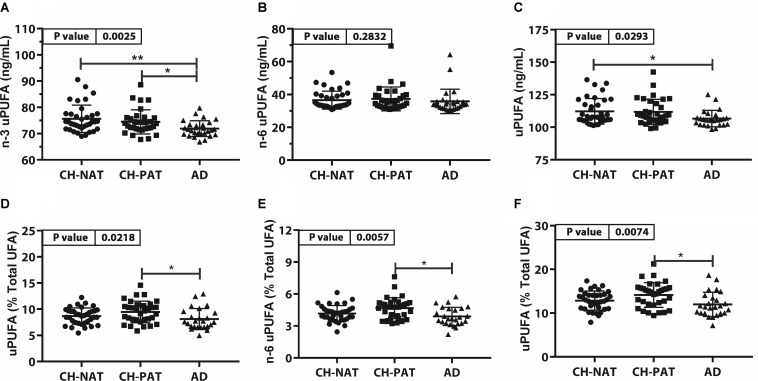
Unesterfied PUFAs (uPUFA) in CH-NAT, CH-PAT, and AD – Scatter plots (mean ± SD) of the concentrations of n-3 uPUFAs **(A)**, n-6 uPUFA **(B)**, and uPUFA **(C)** showing *p*-values determined using 1-way ANOVA and adjusted *p*-values < 0.05 indicated by an asterisk (*). Plots of the proportions of n-3 uPUFAs **(D)**, n-6 uPUFA **(E)**, and uPUFA **(F)** showing *p*-values obtained using 1-way ANOVA and adjusted *p*-values < 0.05 indicated by an asterisk (*).

**FIGURE 5:**
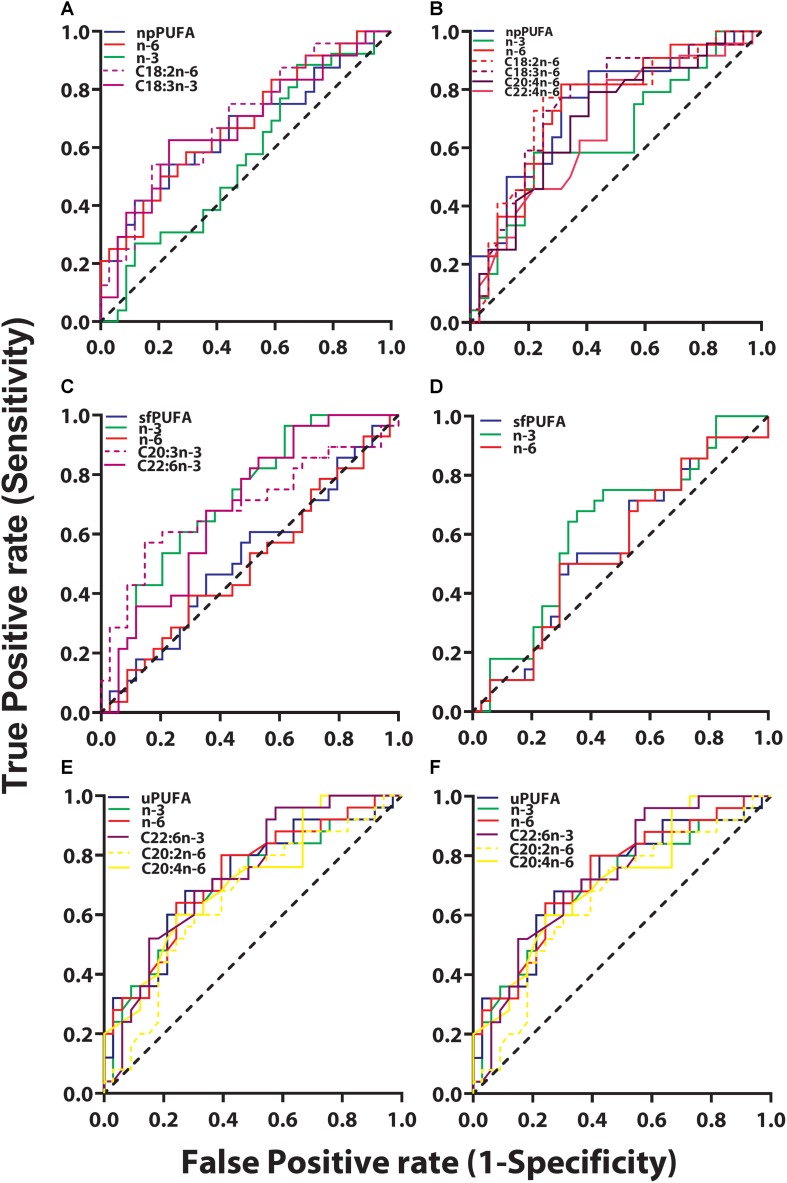
ROC for CH-PAT versus AD – *ROC of PUFA concentrations in the NP fraction (npPUFA)-* The proportion of PUFA (AUC = 0.66, *p* = 0.0344), n-6 PUFA (AUC = 0.68, *p* = 0.0195, C18:3n-6 (AUC = 0.68, *p* = 0.0221), and C18:2n-6 (AUC = 0.69, *p* = 0.0126) were significant classifiers of CH-PAT and AD **(A)**. More significantly is the expression of NP PUFA per billion NPs that showed that PUFAs (AUC = 0.75, *p* = 0.0023), n-6 PUFA (AUC = 0.74, *p* = 0.0031), C22:4n-6 (AUC = 0.67, *p* = 0.0348), C30:4n-6 (AUC = 0.70, *p* = 0.0116), C18:3n-6 (AUC = 0.76, *p* = 0.0015), and C18:2n-6 (AUC = 0.75, *p* = 0.0019) were binary classifiers of CH-PAT and AD **(B)**. *ROC of PUFA concentrations in the SF fraction (sfPUFA)-* For PUFAs in the SF fraction, n-3 PUFA (AUC = 0.72, *p* = 0.0032), C22:6n-3 (AUC = 0.69, *p* = 0.0113), and C20:3n-3 (AUC = 0.69, *p* = 0.0096) **(C)**, were accurate classifiers of CH-PAT and AD, while the proportion of PUFAs in the SF fraction did not significantly classify these clinical groups **(D)**. *ROC for PUFA concentrations in the unesterified fraction (uPUFA).* In the unesterified fractions, levels of PUFA (AUC = 0.70, *p* = 0.0083), n-3 (AUC = 0.68, *p* = 0.0197), C22:6n-3 (AUC = 0.66, *p* = 0.0334), C20:2n-6 (AUC = 0.72, *p* = 0.0046) were binary classifiers of CH-PAT and AD **(E)**. When expressed as a proportion of all fatty acids, PUFA (AUC = 0.70, *p* = 0.0094), n-3 (AUC = 0.72, *p* = 0.0041), n-6 (AUC = 0.73, *p* = 0.0032), C22:6n-3 (AUC = 0.73, *p* = 0.0024), C20:4n-6 (AUC = 0.71, *p* = 0.0059), and C20:2n-6 (AUC = 0.66, *p* = 0.0428) were binary classifiers of CH-PAT and AD **(F)**.

**FIGURE 6:**
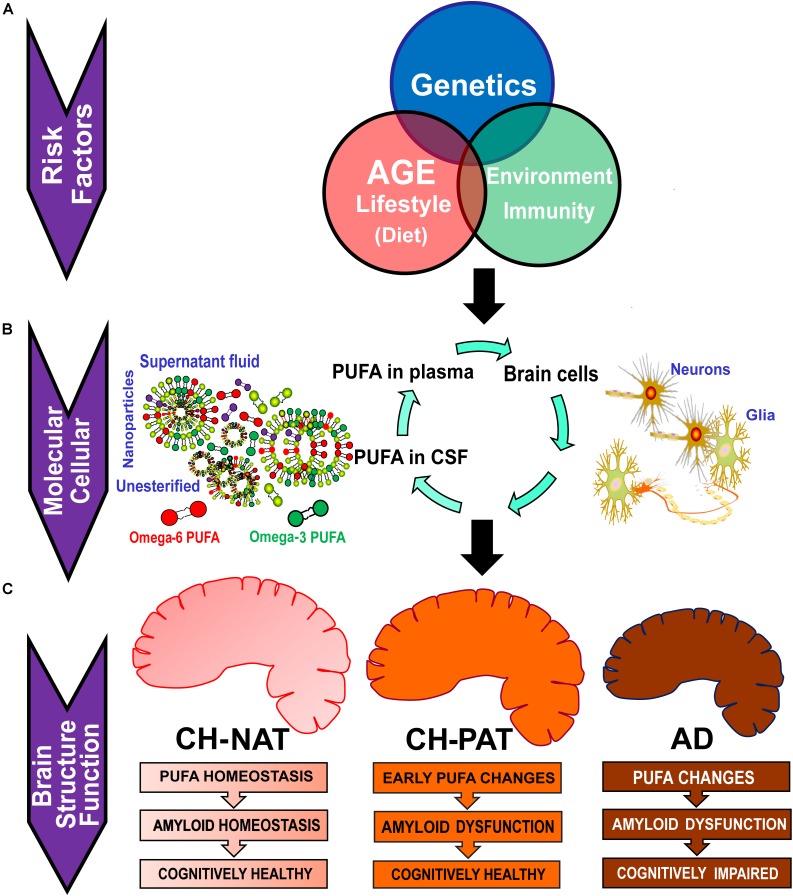
Potential factors that contribute to the dysfunction in PUFA homeostasis in AD. (A) Risk factors such as genetic variants, age, lifestyle, and environmental exposure can influence plasma PUFA metabolism. **(B)** Changes in plasma and CSF PUFA levels are manifested at the molecular and cellular levels. Plasma PUFAs are transported into the brain where they are used for signaling, immune response, or for the energy needs of the brain. CSF PUFA is considered a fair representation of brain metabolism. PUFAs in CSF are found in nanoparticles (npPUFA), in the supernatant fluid (sfPUFA), or as unesterified species (uPUFA). **(C)** In CH-NAT study participants, there is homeostasis in PUFA metabolism resulting in cognitively healthy brain function. In the CH-PAT brain, there are early signs of dysfunction in PUFA metabolism but not significant enough to cause cognitive decline, while in the AD brain, there is dysfunction in PUFA metabolism. Whereas Aβ_42_ levels are similar in CH-PAT and AD, cognitive resilience in CH-PAT is allied with the higher PUFA levels in the brain. We conclude that dysfunction in PUFA homeostasis contributes to neuronal apoptosis that subsequently results in the neuropsychological deficits that characterize AD.

The concentrations of n-3 npPUFA (Figure 2A), n-6 npPUFA (Figure 2B), and npPUFA (Figure 2C) were similar in all clinical groups. However, when expressed as a proportion of all fatty acids in the NF fraction, n-6 npPUFA was significantly lower in AD than in CH-PAT (Figure 2E) while n-3 npPUFA and npPUFA trended lower in AD than in CH-NAT (Figure 2D) and CH-PAT (Figure 2F). Further ANOVA analyses of individual PUFAs showed that four PUFAs (C20:2n-6, C20:3n-3, C22:4n-6, C22:5n-3) were enriched in nanoparticles of AD compared with CH-NAT (Supplementary Table 1).

#### NP Concentration Adjusted for Nanoparticle Counts

Nanoparticle Tracking Analysis showed that the CSF is enriched with billions of nanoparticles. The average number of particles in CH-NAT and CH-PAT was lower than in AD. Levels of n-3 npPUFAs per billion particles trended lower in AD than in CH-NAT and CH-PAT (Figure 2G). N-6 npPUFA and n-3 npPUFA were significantly lower in AD than CH-NAT and CH-PAT (Figures 2H,I, respectively).

#### PUFA Concentration and Composition in the SF Fraction (Figure 3, Supplementary Table 2)

The concentration of n-3 sfPUFA was significantly lower in AD than in CH-NAT and CH-PAT (Figure 3A) while the concentration of n-6 sfPUFA (Figure 3B), and sfPUFA (Figure 3C) were similar in all clinical groups. The proportion of n-3 sfPUFA, n-6 sfPUFA, and sfPUFA was similar in all clinical groups (Figures 3D–F, respectively). Further ANOVA analyses of individual fatty acids showed levels of three PUFAs (C18:3n-6, C20:4n-6, and C22:6n-3) were ranked CH-PAT>CH-NAT>AD (Supplementary Table 2). However, only DHA reached statistical significance with the highest level in CH-PAT compared with AD (Supplementary Table 2).

#### Unesterified PUFA Concentration and Composition (Figure 4, Supplementary Table 3)

The concentration of n-3 uPUFA was significantly lower in AD than in CH-NAT and CH-PAT (Figure 4A), while the concentration of n-6 uPUFA was similar in all clinical groups. Similar to n-3 uPUFA, uPUFA levels were lower in AD compared with CH-NAT and CH-PAT (Figure 4C). The proportions of n-3 uPUFA, n-6 uPUFA, and uPUFA were higher in CH-PAT than in AD (Figures 4D–F, respectively). Of several uPUFAs species quantified in CSF, the general trend was CH-NAT>CH-PAT>AD for C18:2n-6 and C20:4n-6 (Supplementary Table 3).

### Correlation of PUFAs With Aβ_42_ and Tau (Table 3)

To further test our hypothesis that changes in PUFA composition can influence amyloid-beta metabolism, we performed Spearman ranked correlation analyses comparing fatty acids in each CSF fraction with CSF amyloid and T-tau levels in all study participants and in each subgroup. We did not find any correlation between CSF PUFA and Aβ_42_/T-tau in all study participants, probably due to the complex pathology of the three clinical groups. Therefore, we examined the CH, the CH-NAT, and CH-PAT groups separately.

**TABLE 3:** Fatty acids that correlate with CSF Aβ42 or T-tau levels.

CSF fraction	CH	CH-NAT	CH-PAT
**Fatty acid levels that correlate with CSF Aβ_42_**			
NP PUFA	C18:2n-6, (−0.3)*		C18:3n-6, (−0.34)*
	n-6, (−0.25)*		N-6/N-3 npPUFA, (−0.38)*
			AA/DHA, (−0.37)*
			AA/(EPA+DHA), (−0.35)*
Unesterified PUFA		C22:5n-3, (−0.38)*	
		C22:6n-3, (−37)*	
		n-3 uPUFA, (−0.38)*	
		uPUFA, (−0.37)*	
		AA/(EPA+DHA), (0.46)**	
**Fatty acid levels that correlate with CSF T-tau levels**			
NP PUFA		C20:4n-6, (0.38)*	
SF PUFA	C20:4n-6, (0.26)*	C18:2n-6, (−0.38)*	C20:4n-6, (0.35)*
	C22:6n-3, (0.43)***	C22:6n-3, (0.40)*	C22:6n-3, (0.54)**
	n-3, (0.29)*		n-3, (0.48)**

Spearman correlation coefficient (ρ) with *p* < 0.05 (*), *p* < 0.01 (**), and *p* < 0.005 (***).

*CH*– For all CH participants, C18:2n-6 and n-6 npPUFA in the NP fractions negatively correlated with Aβ_42_ levels (Table 3). For the SF PUFAs, C20:4n-6, C22:6n-3, and n-3 positively correlated with Aβ_42_.

*CH-NAT* – No NP PUFAs correlated with Aβ_42_ levels. For uPUFAs, our studies show that three PUFAs (C22:5n-3, C22:6n-3), total PUFA, and n-3 PUFA inversely correlated with Aβ_42_. The ratio C20:4n-6/(C20:5n-3+C22:6n-3) positively correlated with Aβ_42_ levels. C20:4n-6 in NP and C22:6 in SF positively correlated with total T-tau. C18:2n-6 in the SF fraction negatively correlated with T-tau levels.

*CH-PAT*. In the NP fraction, C18:3n-6, n-6/n-3 PUFA ratio, C20:4n-6/C22:6n-3 and C20:4n-6/(C20:5n-3+C22:6n-3) ratios were inversely correlated with CSF Aβ_42_ levels. C20:4n-6, C22:6n-3 and n-3 PUFAs positively correlated with T-tau in the SF fraction.

### ROC Analyses to Determine Fatty Acid Classifiers of Clinical Groups (Figure 5)

ROC analyses were performed between clinical groups to determine if PUFA levels in CSF fractions could differentiate the three clinical groups. Among all PUFAs analyzed, only two were able to classify between CH-NAT and CH-PAT subjects. Both were NP derived n-6 PUFA fatty acids and higher levels classified CH-PAT with AUC of 0.65 (*P* = 0.027) and 0.64 (*P* = 0.042) for C18.2n-6 and C20:4n-6, respectively.

The most striking changes were noticed when ROC analyses were performed for CH-NAT compared with AD. Four (C20:2n-6, C20:3n-3, C20:3n-6, and C22:5n-3) of the nine free PUFAs and levels of n-3 PUFAs were able to perform as binary classifiers of AD. In the SF fraction, two PUFAs (C20:3n-3, C22:6n-3), and levels of N-3 PUFAs performed as binary classifiers of AD subjects. Of the ten NP PUFAs analyzed, five were found to perform as binary classifiers of AD. ROC curve analysis showed that lower levels of C20:5n-3 classified AD while higher levels of C20:3 (n-3), C20:4, C22:4, and C22:5n-3 were shown to be classifiers of AD.

We next examined whether PUFAs in CSF fractions distinguished CH-PAT from AD. None of the PUFAs in the NP fractions were classifiers of CH-PAT/AD (data not shown). However, the proportion of PUFA, n-6 PUFA, C18:3n-6, and C18:2n-6 were significant classifiers of CH-PAT and AD (Figure 5A). More significantly is the expression of NP PUFA per billion NPs that showed that PUFAs (*p* < 0.005), n-6 PUFA (*p* < 0.005), C22:4n-6 (*p* = 0.05), C30:4n-6 (AUC = 0.70, *p* < 0.01), C18:3n-6 (*p* < 0.005), and C18:2n-6 (*p* < 0.005) were binary classifiers of CH-PAT and AD (Figure 5B). For PUFAs in the SF fraction, n-3 PUFA (*p* < 0.005), C22:6n-3 (*p* < 0.05), and C20:3n-3 (*p* < 0.01) (Figure 5C), were accurate classifiers of CH-PAT and AD, while the proportion of PUFAs in the SF fraction did not significantly classify these clinical groups (Figure 5D). In the unesterified fractions, levels of PUFA, n-3, C22:6n-3, and C20:2n-6 were binary classifiers of CH-PAT and AD (Figure 5E). When expressed as a proportion of all unesterified fatty acids, PUFA, n-3, n-6, C22:6n-3, C20:4n-6, and C20:2n-6 were binary classifiers of CH-PAT and AD (Figure 5F).

## Discussion

As a neurodegenerative disease, AD is irreversible and progresses over many years before symptoms are evident (Filley, 1995). While CSF Aβ_42_ is recognized as an early indicator of AD, it is known that this pathology can present for several decades before the onset of clinical symptoms, while p-Tau levels are closely linked with symptoms (Wallin et al., 2006). We identified a group of cognitively normal subjects with CSF Aβ_42_ similar to symptomatic AD (Harrington et al., 2013). Given that amyloid precursor protein (APP) is a membrane-bound protein, we hypothesize that changes in membrane lipid components will influence APP processing, and these changes are potential early indicators of AD. To test this hypothesis, we examined fatty acid levels in three clinical groups; our CH population, into a group with normal Aβ_42_ and T-tau ratio (CH-NAT) and a second group with pathological Aβ_42_/T-tau ratio (CH-PAT) similar to AD subjects. The aim of our study was to determine if PUFA differences in CSF fractions can classify these subgroups and isolate CH-PAT from AD that has similar CSF Aβ_42_/Tau ratios. The major findings of our study include: (1) No differences in plasma LDL and HDL, and no significant differences in self-reported consumption of fatty acids between the three clinical groups. (2) Significant differences in some PUFA levels in CSF fractions between our three clinical groups. (3) The progressive decrease in the amount of PUFA per billion nanoparticles in CH-NAT to CH-PAT to AD. (4) Correlation of some PUFA species with CSF Aβ_42_ or with CSF T-tau. (5). ROC analyses showing that some CSF PUFAs can perform as effective binary classifiers of our clinical subgroups. The implications of our study are that early changes in brain PUFA compositions are reliable indicators of AD pathology. Importantly, PUFAs contribute to the cognitive resilience in our pre-symptomatic AD population.

Figure 6 illustrates the factors that may influence PUFA metabolism and the clinical consequences of such changes. Several risk factors acting independently or interacting with each other can influence plasma PUFA metabolism, resulting in changes in plasma and CSF PUFA levels. Our studies show changes in PUFA species in CSF fractions that may be representative of changes in brain cells. The three different fractions have different PUFA composition and differentially affected by AD pathology. There is a similarity in the PUFA composition of nanoparticles (npPUFA) and with unesterified species (uPUFA). These data suggest that membrane-bound phospholipases that have been shown to localize with amyloid plaques may be involved in the release of uPUFA from npPUFA. Considering differences in our clinical groups, CH-NAT versus AD represented the greatest changes in PUFA. We also noticed that DHA was the most consistent difference between CH-PAT and AD, suggesting that the cognitive resilience shown by the CH-PAT participants may be attributed to the higher DHA concentrations in their fractions. Overall, CH-NAT study participants display homeostasis in PUFA metabolism and are, therefore, cognitively healthy. In CH-PAT, there are early signs of dysfunction in PUFA metabolism, while in the AD brain, there is dysfunction in PUFA metabolism. Dysfunction in PUFA metabolism contributes to neuronal apoptosis that subsequently results in the neuropsychological deficits that characterize AD.

Since LDL/HDL levels and self-reported fatty acid consumption are similar in our clinical groups, it implies that changes we observe in CSF fractions are due to transport to the brain or *in vivo* metabolism of fatty acids. Of all fatty acids detected, we found significant changes in several PUFAs, including AA (C20:4n-6) and DHA (C22:6n-3). These changes in PUFA metabolism may play an important role in the progression of AD pathology by not only influencing Aβ_42_ formation but also affecting mitochondrial energy homeostasis and the generation of inflammatory or pro-resolving and immune modulators.

### PUFA and Cognitive Function

PUFAs influence cognitive outcomes with several studies showing that n-3 supplementation improved cognitive measurements (Chiu et al., 2008). Changes in n-3 PUFAs levels in red blood cells are linked to visual memory, executive function, and abstract thinking (Tan et al., 2012). Another study found that lower n-6 to n-3 ratios predicted executive function with adolescents performing better on tests of cognitive function and holding shorter processing times on memory tasks (Sheppard and Cheatham, 2013), In contrast, increased inflammatory n-6 cascade results in heightened IL-1β and a subsequent decline in working memory in a rodent model (Matsumoto et al., 2004). N-3 PUFAs are known to influence brain structure by increasing synaptic protein expression leading to increases in numbers of c-Fos-positive neurons and hippocampal neurogenesis (Wang et al., 2010). Our study shows that n-6 and n-3 fatty acids inversely and directly correlate with Aβ_42_ or with T-tau, respectively. In addition, whereas Aβ_42_ levels are similar in CH-PAT and AD, several PUFAs in CSF fractions are significantly lower in AD than in CH-PAT. While many n-3 fatty acids decreased, higher levels of two NP n-6 FAs (C20:4n-6 and C22:4n-6) classified AD participants (Figure 5). These data suggest that PUFA changes are closely linked to cognitive resilience in our CH-PAT population than Aβ_42_ levels. However, the inverse correlation of PUFA with Aβ_42_ suggests there is an association between PUFA and the clearance of neurotoxic Aβ_42_ from the brain. Thus, changes in different PUFA types and ratios may impact brain structure and result in cognitive changes associated with AD pathology.

### PUFA and Aβ_42_

We recently showed differences in fatty acid composition in CSF fractions from CH, MCI, and AD individuals (Fonteh et al., 2014). Fatty acyls incorporated into glycerophospholipids or sphingolipids are major components of the cell membrane of brain cells. These membrane lipids create the appropriate environment for ion channels, receptors, structural proteins, and transmembrane proteins, including APP, needed for the proper functioning of brain cells. Disruption of membrane components, including fatty acid composition, can alter interactions with these proteins. For example, physical measurements show that the ratio of saturated to PUFA influences how Aβ_42_ binds to BACE (Cole and Frautschy, 2006; Grimm et al., 2011; Eto et al., 2019; Marwarha et al., 2019). Similar changes in fatty acids that we measured in CSF fractions, and their correlation with Aβ_42_ and T-tau in our clinical groups confirm the role of fatty acids in AD severity and progression.

Fatty acids are also involved in posttranslational modification of membrane proteins. The palmitoylation of APP is known to influence its processing (Bhattacharyya et al., 2016). We also found that DHA is higher CH than in AD, free DHA negatively correlates with Aβ_42_ in CH-NAT, and positively correlates with T-tau in SF from CH-NAT and CH-PAT. Given studies showing the non-amyloidogenic and the anti-amyloidogenic role of DHA and its product, neuroprotectin D1 (Sahlin et al., 2007; Eckert et al., 2011; Grimm et al., 2011; Zhao et al., 2011; Grimm et al., 2016), these changes in DHA and other PUFAs in CSF fraction could determine APP processing and changes in cognitive performance of our study population.

### PUFA, Inflammation, and Oxidative Stress

Modifications of PUFAs by enzyme oxidation or auto-oxidation can alter the physical properties of neuronal membranes, generate inflammatory mediators, or pro-resolving anti-inflammatory mediators (Qu et al., 2015; Bazan et al., 2017; Poreba et al., 2017). Auto-oxidation forms isoprostanes that are indicators of oxidative stress and are neurotoxic. On the other hand, resolvins and neuroprotectins resolve inflammation and are involved in the repair of post-mitotic brain cells (Heras-Sandoval et al., 2016; Ho et al., 2018). Therefore, a balance in the levels of n-6 to n-3 levels can impact neuronal function and survival. The importance of DHA is revealed by studies showing that supplementation showed a correlative relationship with immunoregulation in AD (Freund-Levi et al., 2014). Similarly, hematocytes treated with EPA have reduced IL-1β/IL-10 ratio and IL-6/IL-10 ratio (Serini et al., 2012). This anti-inflammatory role for n-3 PUFA is counteracted by the inflammatory effects of n-6 PUFA AA. AA metabolites are associated with cellular redox increase of Cox-2 expression, while n-3 PUFA decreases Cox-2 expression (Gravaghi et al., 2011; Mitjavila and Moreno, 2012). Several studies have documented increased oxidative stress in AD brain (Butterfield et al., 1999; Ansari and Scheff, 2010; Bonda et al., 2010; Mosconi et al., 2008; Raukas et al., 2012; Lee et al., 2013; Eckman et al., 2018) typified by increases in reactive oxygen species (ROS). ROS contribute to neurodegeneration and atrophy in neurites (Munnamalai and Suter, 2009). N-3 PUFAs have been found to lower oxidative stress and impact aging (Kiecolt-Glaser et al., 2013). In agreement with these studies, we report a decrease in DHA and an increase in AA in NP of CH-PAT compared with AD study participants. Moreover, several PUFAs are negatively or positively correlated with Aβ42 and T-tau levels in CSF, respectively (Table 3). These data suggest that dysregulation of PUFA metabolism occurs in preclinical AD and may be linked with a balance in inflammatory and anti-inflammatory signaling pathways. Inflammatory pathways in CH-PAT are counteracted by higher n-3 fatty acids in CSF fractions compared with AD subjects. N-3 to n-6 PUFA homeostasis may determine the inflammatory capacity of the brain with higher or lower n-3 to n-6 ratio signaling protection or progression from pre–symptomatic to symptomatic AD, respectively.

### PUFA and Brain Energy

It is now recognized that neurological disorders are associated with metabolic syndromes (Farooqui et al., 2012; Lucke-Wold et al., 2014). The brain’s principal normal source of energy is from sugars, but liver-derived ketone bodies become a relevant source of brain energy during fasting (Zhang et al., 2013; Wu et al., 2018). In AD, ketogenic diets do not only provide an energy source to mitigate oxidative damage associated with metabolic stress but may be crucial in mitochondrial biogenesis. Recently, ketone bodies have been shown to have an inflammasome activity (Neudorf et al., 2019). Thus, changes in ketone body precursor fatty acids that we measure in CSF may reflect metabolic stress in the brain associated with AD. Dysfunctional brain energy may impact repair of post-mitotic neuronal membranes and clearance of neurotoxic or damaged macromolecules such as Aβ_42_. Although there is an increase in inflammatory n-6 fatty acids in CH-PAT, this is counteracted by increases in n-3 PUFAs that maintain pro-resolving mediators in the brain. This process protects the CH-PAT brain and maintains cognitive function, although Aβ_42_ levels would indicate otherwise.

## Conclusion

These studies support our hypothesis that brain PUFA metabolism is altered in the early phases of AD. The balance in fatty acid metabolism in CSF fractions when comparing cognitively normal subjects with normal and abnormal amyloid to the T-tau ratio shows that PUFA metabolism is important in the clinical progression of dementia. We propose that dietary approaches that maintain normal PUFA levels, especially supplementation studies that favor pro-resolving fatty acids over inflammatory fatty acids may enhance cognitive function in an elderly population. The use of antioxidants or regulators of PUFA metabolism may maintain n-3 to n-6 balance and prevent cognitive decline in aging seniors. Further longitudinal studies to determine the rate and causes of PUFA changes are required to reveal novel biomarkers and validate potential preventive approaches.

## Data Availability Statement

The datasets generated for this study are available on request to the corresponding author.

## Ethics Statement

The studies involving human participants were reviewed and approved by the Huntington Memorial Hospital, Pasadena, California IRB #HMH-99-09. The patients/participants provided their written informed consent to participate in this study.

## Author Contributions

AF and MH contributed to the conceptualization and study design, writing of original draft and manuscript preparation, project administration, resources, and funding acquisition. AF, MH, MC, AC, and SE contributed to the methodology. AF contributed to the validation and supervision. AC, SE, XA, and AF contributed to the formal analysis. AC, MC, and AF contributed to the data curation. XA, MH, and AF contributed to the manuscript review and editing.

## Conflict of Interest

The authors declare that the research was conducted in the absence of any commercial or financial relationships that could be construed as a potential conflict of interest.
