# Supplementary material for: Polyunsaturated Fatty Acid Composition of Cerebrospinal Fluid Fractions Shows Their Contribution to Cognitive Resilience of a Pre-symptomatic Alzheimer’s Disease Cohort
Source: Front Physiol. 2020 Feb 14;11:83. doi: 10.3389/fphys.2020.00083 (PMC7034243; doi:10.3389/fphys.2020.00083)
Supplement: Supplementary file 1 [file Table_1.docx]

**Supplementary Tables**

**Supplementary Table 1A.** PUFA levels in brain-derived nanoparticles from CH-NAT, CH-PAT, and AD study participants

| Fatty acids | Levels in CH-NAT(ng/mL)  Mean ± SEM (95 % CI)  n=36 | Levels in CH-PAT (ng/mL)  Mean ± SEM (95 % CI)  n=34 | Levels in AD (ng/mL)  Mean ± SEM (95 % CI)  n=25 |
| --- | --- | --- | --- |
| C18:2n-6 | 18.8 ± 2.5 (12.7-23.0) | 18.7 ± 1.3 (16.1-21.3) | 20.8 ± 5.0 (10.5-31.2) |
| C18:3n-6 | 47.8 ± 7.1 (33.4-62.2) | 46.1 ± 4.3 (37.4-54.8) | 51.8 ± 14.9 (21.0-82.7) |
| C20:2n-6*^α^ | 5.4 ± 0.2 (5.1–5.8) | 5.7 ± 0.2 (5.3–6.0) | 6.6 ± 0.5 (5.5–7.7) |
| Dihomo-γ-C20:3n-6 | 1.0 ± 0.1 (0.8-1.2) | 1.1 ± 0.1 (0.9-1.3) | 1.0 ± 0.1 (0.8-1.2) |
| C20:4n-6 | 6.7 ± 0.3 (6.0-7.3) | 7.8 ± 0.5 (6.9-8.8) | 7.6 ± 0.4 (6.7-8.5) |
| C22:4n-6***^α^ | 3.8 ± 0.1 (3.6 – 4.0) | 4.0 ± 0.1 (3.9 – 4.4) | 4.6 ± 0.3 (4.0–5.2) |
| C20:3n-3**^αβ^ | 11.5 ± 0.3 (10.8-12.1) | 12.0 ± 0.4 (11.2-12.8) | 14.3 ± 1.2 (11.9-16.7) |
| C20:5n-3 | 1.8 ± 0.2 (1.3-2.2) | 1.6 ± 0.3 (1.4-2.6) | 1.6 ± 0.4 (0.7-2.5) |
| C22:5n-3**^α^ | 7.7 ± 0.2 (7.2 – 8.2) | 8.1 ± 0.3 (7.5 – 8.6) | 9.6 ± 0.8 (8.0–11.3) |
| C22:6n-3 | 60.1 ± 10.8 (38.2-82.0) | 57.8 ± 10.1(37.2-6-78.4) | 43.9 ± 6.5 (30.5-57.3) |

Abbreviations: CI, confidence interval; D-γ-, Dihomo gamma

* *p < 0.05, ** p < 0.01, ***p < 0.005* by ANOVA. Tukey’s Multiple Comparison Test: ^α^ *p < 0.05* for CH-NAT versus AD; ^β^ *p < 0.05* for CH-PAT versus AD.

**Supplementary Table 1B. npPUFA** composition of brain-derived nanoparticles from CH-NAT, CH-PAT, and AD study participants

| Fatty acids | Levels in CH-NAT (%)  Mean ± SEM (95 % CI)  n=36 | Levels in CH-PAT (%)  Mean ± SEM (95 % CI)  n=34 | Levels in AD (%)  Mean ± SEM (95 % CI)  n=25 |
| --- | --- | --- | --- |
| C18:2n-6 | 0.53 ± 0.05 (0.42-0.63) | 0.54 ± 0.10 (0.34-0.73) | 0.51 ± 0.15 (0.21-0.81) |
| C18:3n-6 | 1.33 ± 1.08 (0.98-1.68) | 1.46 ± 0.26 (0.92-2.0) | 2.31 ± 1.08 (0.09-4.53) |
| C20:2n-6 | 0.16 ± 0.01 (0.14–0.18) | 0.16 ± 0.01 (0.14–0.18) | 0.15 ± 0.01 (0.13–0.16) |
| Dihomo-γ-C20:3n-6 | 0.03 ± 0.01 (0.02–0.04) | 0.03 ± 0.01 (0.02–0.04) | 0.03 ± 0.01 (0.02–0.04) |
| C20:4n-6 | 0.21 ± 0.01 (0.18–0.24) | 0.19 ± 0.01 (0.17–0.20) | 0.18 ± 0.01 (0.16–0.20) |
| C22:4n-6 | 0.11 ± 0.01 (0.10-0.13) | 0.11 ± 0.01 (0.10-0.12) | 0.11 ± 0.01 (0.10-0.12) |
| C20:3n-3 | 0.33 ± 0.02 (0.29-0.38) | 0.33 ± 0.02 (0.30-0.36) | 0.33 ± 0.02 (0.30-0.37) |
| C20:5n-3 | 0.05 ± 0.01 (0.03-0.07) | 0.05 ± 0.01 (0.04-0.07) | 0.04 ± 0.01 (0.02-0.05) |
| C22:5n-3 | 0.22 ± 0.01 (0.20-0.25) | 0.22 ± 0.01 (0.20-0.24) | 0.22 ± 0.01 (0.20-0.24) |
| C22:6n-3 | 1.34 ± 0.22 (0.90-1.78) | 1.64 ± 0.30 (1.03-2.25) | 1.16 ± 0.19 (0.78-1.55) |

**Supplementary Table 2A:** PUFA levels of supernatant fluid from CH-NAT, CH-PAT and AD study participants

|  | Levels in CH-NAT, n=35 | Levels in CH-PAT, n=34 | Levels in AD-PAT, n=25 |
| --- | --- | --- | --- |
| Fatty acids | ng/mL, Mean ± SEM, (95 % CI) | | |
| C18:2n-6 | 736.2 ± 65.2 (603.8-868.7) | 694.9 ± 59.4 (574-815.8) | 788.7 ± 88.8 (605.6-971.9) |
| C18:3n-6 | 843.7 ± 75.8 (689.6-997.8) | 947.6 ± 176.3 (589-1306) | 845.4 ± 96.5 (646.2–1045) |
| Dihomo-γ-C20:3n-6 | 29.6 ± 5.3 (18.9-40.3) | 27.1 ± 2.9 (21.3- 32.9) | 23.6 ± 2.7 (18.0-29.2) |
| C20:4n-6 | 516.9 ± 46.7 (421.9-611.9) | 551.3 ± 54.7 (440.1-662.5) | 515.2 ± 55.9 (399.9 – 630.5) |
| α-C18:3n-3 | 22.3 ± 2.6 (17.1-27.5) | 22.5 ± 2.9 (16.6-28.4) | 20.0 ± 1.2 (17.6-22.4) |
| C20:3n-3 | 35.0 ± 1.64 (31.7-38.4) | 33.2 ± 0.2 (32.8-33.5) | 32.6 ± 0.2 (32.2-33.0) |
| C20:5n-3 | 9.7 ± 1.6 (6.5-12.9) | 9.9 ± 1.7 (6.5-13.2) | 5.9 ± 0.8 (4.3-7.4) |
| C22:6n-3*^α^ | 256.4 ± 15.8 (224.4-288.5) | 270.2 ± 18.8 (232-308.5) | 202.2 ± 15.3 (170.6-233.9) |

Abbreviations: CI, confidence interval; D-γ-, Dihomo gamma

* *p < 0.05* ANOVA. Tukey’s Multiple Comparison Test: ^α^ *p < 0.05* for CH-PAT versus AD.

**Supplementary Table 2B:** PUFA composition of supernatant fluid from CH-NAT, CH-PAT and AD study participants

|  | Levels in CH-NAT, n=35 | Levels in CH-PAT, n=34 | Levels in AD-PAT, n=25 |
| --- | --- | --- | --- |
| Fatty acids | % of Total fatty acids, Mean ± SEM, (95 % CI) | | |
| C18:2n-6 | 2.88 ± 0.33 (2.2-3.56) | 2.94 ± 0.36 (2.2-3.67) | 2.95 ± 0.53 (1.86-4.03) |
| C18:3n-6 | 2.60 ± 0.15 (2.30-2.90) | 2.51 ± 0.17 (2.17-2.86) | 3.22 ± 0.95 (1.26-5.17) |
| Dihomo-γ-C20:3n-6 | 0.15 ± 0.03 (0.09-0.20) | 0.17 ± 0.03 (0.10-0.24) | 0.13 ± 0.04 (0.06-0.29) |
| C20:4n-6 | 1.90 ± 0.21 (1.48-2.32) | 2.08 ± 0.23 (1.61-2.56) | 1.65 ± 0.18 (1.28-2.03) |
| α-C18:3n-3 | 0.09 ± 0.01 (0.07-0.11) | 0.10 ± 0.01 (0.07-0.12) | 0.08 ± 0.01 (0.06-0.11) |
| C20:3n-3 | 0.17 ± 0.03 (0.12-0.22) | 0.17 ± 0.03 (0.12-0.22) | 0.15 ± 0.03 (0.09-0.22) |
| C20:5n-3 | 0.07± 0.02 (0.04-0.14) | 0.09± 0.01 (0.04-0.09) | 0.04± 0.01 (0.02-0.06) |
| C22:6n-3*^α^ | 1.05 ± 0.13 (0.79-1.30) | 1.16 ± 0.17 (0.82-1.49) | 0.76 ± 0.11 (0.54-0.99) |

**Supplementary Table 3A:** Unesterified PUFA levels in CSF from CH-NAT, CH-PAT, and AD study participants

| Free Fatty acids | Levels in CH-NAT  Mean ± SEM (95 % CI)  (ng/ml) n=36 | Levels in CH-PAT  Mean ± SEM (95 % CI)  (ng/ml) n=33 | Levels in AD  Mean ± SEM (95 % CI)  (ng/ml) n=25 |
| --- | --- | --- | --- |
| C18:2n-6 | 2.1 ± 0.6 (1.0-3.3) | 2.9 ± 1.1 (0.7-5.1) | 1.3 ± 0.8 (-0.5-3.0) |
| C20:2n-6 | 15.5 ± 0.1 (15.4-15.7) | 15.5 ± 0.1 (15.3-15.6) | 15.3 ± 0.2 (15.0-15.6) |
| Dihomo−γ-C20:3n-6 | 1.4 ± 0.1 (1.2-1.7) | 1.3 ± 0.1 (1.0-1.5) | 1.2 ± 0.1 (0.9-1.4) |
| C20:4n-6 | 9.5 ± 0.3 (8.9-10.1) | 9.7 ± 0.4 (8.9-10.4) | 9.1 ± 0.3 (8.5-9.7) |
| C22:4n-6 | 8.0 ± 0.1 (7.9-8.1) | 8.0 ± 0.1 (7.9-8.1) | 8.0 ± 0.1 (7.9-8.3) |
| C20:3n-3 | 34.6 ± 0.3 (34.0-35.3) | 34.1 ± 0.3 (33.6-34.6) | 33.7 ± 0.3 (33.1-34.4) |
| C20:5n-3 | 2.1 ± 0.5 (1.0-3.1) | 1.8 ± 0.5 (0.8-2.8) | 1.1 ± 0.2 (0.6-1.6) |
| C22:5n-3 | 22.0 ± 0.04 (22.0-22.1) | 22.0 ± 0.04 (21.9-22.1) | 22.0 ± 0.1 (21.9-22.1) |
| C22:6n-3*^α^ | 17.0 ± 0.5 (15.9-18.0) | 16.6 ± 0.6 (15.4-17.8) | 15.0 ± 0.4 (14.2-15.7) |

Abbreviations: CI, confidence interval; D-γ-, Dihomo gamma

** p < 0.05, ** p < 0.01, ***p < 0.005* by ANOVA. Tukey’s Multiple Comparison Test: ^α^ *p < 0.05* for CH_NAT versus AD, ^β^ *p < 0.05* for CH-PAT versus AD.

**Supplementary Table 3B:** Unesterified PUFA composition in CSF from CH-NAT, CH-PAT, and AD study participants

| Free Fatty acids | Levels in CH-NAT  Mean ± SEM (95 % CI)  (% Total) n=36 | Levels in CH-PAT  Mean ± SEM (95 % CI)  (% Total) n=33 | Levels in AD  Mean ± SEM (95 % CI)  (% Total) n=25 |
| --- | --- | --- | --- |
| C18:2n-6 | 0.50 ± 0.09 (0.30-0.69) | 0.58 ± 0.20 (0.16-1.0) | 0.43 ± 0.26 (-0.21-1.07) |
| C20:2n-6 | 1.79 ± 0.06 (1.67-1.9) | 1.98 ± 0.08 (1.80-2.15) | 1.73 ± 0.09 (1.55-1.92) |
| Dihomo−γ-C20:3n-6 | 0.16 ± 0.01 (0.14-0.18) | 0.15 ± 0.01 (0.13-0.18) | 0.13 ± 0.01 (0.11-0.16) |
| C20:4n-6 | 1.08 ± 0.03 (1.01-1.15) | 1.20 ± 0.05 (1.11-1.30) | 1.01 ± 0.04 (0.93-1.08 |
| C22:4n-6 | 0.92 ± 0.03 (0.86-0.96) | 1.02 ± 0.04 (0.94-1.10) | 0.90 ± 0.04 (0.82-0.98) |
| C20:3n-3 | 4.0 ± 0.14 (3.72-4.27) | 4.38 ± 0.19 (3.99-4.76) | 3.83 ± 0.21 (3.39-4.26) |
| C20:5n-3 | 0.22 ± 0.06 (0.09-0.34) | 0.28 ± 0.11 (0.08-0.30) | 0.13 ± 0.03 (0.07-0.19) |
| C22:5n-3 | 2.53 ± 0.07 (2.38+2.69) | 2.81 ± 0.12 (2.58+3.05) | 2.48 ± 0.12 (2.23+2.73) |
| C22:6n-3 | 1.93 ± 0.07 (1.79+2.08) | 2.09 ± 0.09 (1.90+2.28) | 1.67 ± 0.07 (1.53+1.82) |
